# Roots and Tuber Crops as Functional Foods: A Review on Phytochemical Constituents and Their Potential Health Benefits

**DOI:** 10.1155/2016/3631647

**Published:** 2016-04-03

**Authors:** Anoma Chandrasekara, Thamilini Josheph Kumar

**Affiliations:** Department of Applied Nutrition, Wayamba University of Sri Lanka, Makandura, 60170 Gonawila, Sri Lanka

## Abstract

Starchy roots and tuber crops play a pivotal role in the human diet. There are number of roots and tubers which make an extensive biodiversity even within the same geographical location. Thus, they add variety to the diet in addition to offering numerous desirable nutritional and health benefits such as antioxidative, hypoglycemic, hypocholesterolemic, antimicrobial, and immunomodulatory activities. A number of bioactive constituents such as phenolic compounds, saponins, bioactive proteins, glycoalkaloids, and phytic acids are responsible for the observed effects. Many starchy tuber crops, except the common potatoes, sweet potatoes, and cassava, are not yet fully explored for their nutritional and health benefits. In Asian countries, some edible tubers are also used as traditional medicinal. A variety of foods can be prepared using tubers and they may also be used in industrial applications. Processing may affect the bioactivities of constituent compounds. Tubers have an immense potential as functional foods and nutraceutical ingredients to be explored in disease risk reduction and wellness.

## 1. Introduction 

Starchy root and tuber crops are second only in importance to cereals as global sources of carbohydrates. They provide a substantial part of the world's food supply and are also an important source of animal feed and processed products for human consumption and industrial use. Starchy roots and tubers are plants which store edible starch material in subterranean stems, roots, rhizomes, corms, and tubers and are originated from diversified botanical sources. Potatoes and yams are tubers, whereas taro and cocoyams are derived from corms, underground stems, and swollen hypocotyls. Cassava and sweet potatoes are storage roots and canna and arrowroots are edible rhizomes. All these crops can be propagated by vegetative parts and these include tubers (potatoes and yams), stem cuttings (cassava), vine cuttings (sweet potatoes), and side shoots, stolons, or corm heads (taro and cocoyam).

The contribution of roots and tubers to the energy supply in different populations varies with the country. The relative importance of these crops is evident through their annual global production which is approximately 836 million tonnes [1]. Asia is the main producer followed by Africa, Europe, and America. Asian and African regions produced 43 and 33%, respectively, of the global production of roots and tubers [1]. A number of species and varieties are consumed but cassava, potatoes, and sweet potatoes consist of 90% global production of root and tuber crops [1].

Nutritionally, roots and tubers have a great potential to provide economical sources of dietary energy, in the form of carbohydrates (Table 1). The energy from tubers is about one-third of that of an equivalent weight of rice or wheat due to high moisture content of tubers. However, high yields of roots and tubers give more energy per land unit per day compared to cereal grains [2]. In general the protein content of roots and tubers is low ranging from 1 to 2% on a dry weight basis [2]. Potatoes and yams contain high amounts of proteins among other tubers. Sulphur-containing amino acids, namely, methionine and cystine, are the limiting ones in root crop proteins. Cassava, sweet potatoes, potatoes, and yam contain some vitamin C and yellow varieties of sweet potatoes, yam, and cassava contain *β*-carotene. Taro is a good source of potassium. Roots and tubers are deficient in most other vitamins and minerals but contain significant amounts of dietary fibre [2]. Similar to other crops, nutritional value of roots and tubers varies with variety, location, soil type, and agricultural practices, among others.

The burden of noncommunicable diseases (NCDs) increases globally in both developed and developing countries and plays a pivotal role as the major cause of death. Oxidative stress which would be harbored by both endogenous and exogenous factors contributes immensely to the etiology of NCDs as well as the aging process. The association between plant food intake and reduced NCDs episodes has been the main focus of a number of scientific investigations in the recent past. Furthermore, identification of specific plant constituents which convey health benefits is of much interest. Foods of plant origin consist of a wide range of nonnutrient phytochemicals. They are synthesized as secondary metabolites and serve a wide range of ecological roles in home plants [3]. Tubers and root crops are significant sources of a number of compounds, namely, saponins, phenolic compounds, glycoalkaloids, phytic acids, carotenoids, and ascorbic acid. Several bioactivities, namely, antioxidant, immunomodulatory, antimicrobial, antidiabetic, antiobesity, and hypocholesterolemic activities, among others, are reported for tubers and root crops.

This review focuses on the bioactivities of phytochemicals and their distribution in starchy roots and tuber crops. Furthermore, the effect of processing on bioactive compounds of roots and tubers is also discussed.

## 2. Roots and Tuber Crops

Plants producing starchy roots, tubers, rhizomes, corms, and stems are important to nutrition and health. They play an essential role in the diet of populations in developing countries in addition to their usage for animal feed and for manufacturing starch, alcohol, and fermented foods and beverages.

Roots and tuber crops are important cultivated staple energy sources, second to cereals, generally in tropical regions in the world. They include potatoes, cassava, sweet potatoes, yams, and aroids belonging to different botanical families but are grouped together as all types produce underground food. An important agronomic advantage of root and tuber crops as staple foods is their favourable adaptation to diverse soil and environmental conditions and a variety of farming systems with minimum agricultural inputs. In addition, variations in the growth pattern and adopting cultural practices make roots and tubers specific in production systems. However, roots and tuber crops are bulky in nature with high moisture content of 60–90% leading them to be associated with high transportation cost, short shelf life, and limited market margin in developing countries even where they are mainly cultivated.  Table 2 presents commonly consumed starchy tuber and root crops worldwide.

### 2.1. Potatoes (*Solanum tuberosum*)

Potato is currently the fourth most important food crop in the world after maize, wheat, and rice, with a production of 368 million tonnes [1]. It ranks the third after rice and wheat in terms of consumption. Potato is a crop of highland origin and has been domesticated in the high Andes of South America and has become a major food crop in the cool highland areas of South America, Asia, and Central and Eastern Africa [4].

In developed countries potatoes play a pivotal role in the diet compared to those of the developing ones. The energy intake from potatoes by an individual in developed and developing countries was 130 and 41 kcal/day, respectively [5]. Potatoes provide significant amounts of carbohydrates, potassium, and ascorbic acid in the diet [6]. Furthermore, they contribute to 10% of the total folate intake in some European countries, such as Netherlands, Norway, and Finland [7]. In addition, ascorbic acid present in potatoes protects folates from oxidative breakdown [8]. About 50% of the recommended dietary allowance (RDA) of vitamin A may be provided by 250 g of genetically carotenoid enriched potatoes [9]. Potatoes have several secondary metabolites which demonstrated antioxidant as well as other bioactivities [10].

### 2.2. Sweet Potatoes (*Ipomoea batatas* L.)

The origin of sweet potato is Central America, but at present it is widely grown in many tropical and subtropical countries in different ecological regions. It is the seventh largest food crop, grown in tropical, subtropical, and warm temperate regions in the world [11]. Sweet potato can be grown all around the year under suitable climatic conditions and complete crop loss under adverse climatic conditions is rare; thus it is considered as an “insurance crop.” The crop is particularly important in Southeast Asia, Oceania, and Latin America regions and China claims about 90% of total world production. Sweet potatoes are considered as a typical food security crop for disadvantaged populations as the crop can be harvested little by little over a long period of time. National Aeronautics and Space Administration (NASA) has selected sweet potatoes as a candidate crop to be grown and incorporated into the menus for astronauts on space missions due to their unique features and nutritional value [12]. The consumption of 125 g orange fleshed sweet potatoes, rich in carotenoids, improves vitamin A status of children, especially in developing countries [13]. In addition, sweet potatoes are rich in dietary fibre, minerals, vitamins, and bioactive compounds such as phenolic acids and anthocyanins, which also contribute to the color of the flesh.

### 2.3. Cassava (*Manihot esculenta*)

Cassava is the most widely cultivated root crop in the tropics and because of long growth season (8–24 months), its production is limited to the tropical and subtropical regions in the world. Cassava is a perennial shrub belonging to the family Euphorbiaceae. The genus* Manihot* comprises 98 species and* M. esculenta* is the most widely cultivated member [14]. Cassava originated in South America and subsequently was distributed to tropical and subtropical regions of Africa and Asia [15]. Cassava plays an important role as staple for more than 500 million people in the world due to its high carbohydrate content [15]. A number of bioactive compounds, namely, cyanogenic glucosides such as linamarin and lotaustralin, noncyanogenic glucosides, hydroxycoumarins such as scopoletin, terpenoids, and flavonoids, are reported in cassava roots [15–17].

### 2.4. Yams (*Dioscorea* sp.)

Yam is a member of the monocotyledonous family Dioscoreaceae and is a staple food in West Africa, Southeast Asia, and the Caribbean regions [18]. Yam is consumed as raw yam, cooked soup, and powder or flour in food preparations. Yam tubers have various bioactive components, namely, mucin, dioscin, dioscorin, allantoin, choline, polyphenols, diosgenin, and vitamins such as carotenoids and tocopherols [19, 20]. Mucilage of yam tuber contains soluble glycoprotein and dietary fibre. Several studies have shown hypoglycemic, antimicrobial, and antioxidant activities of yam extracts [21, 22]. Yams may stimulate the proliferation of gastric epithelial cells and enhance digestive enzyme activities in the small intestine [23].

### 2.5. Aroids

Aroids are tuber or underground stem bearing plants belonging to the family Araceae. There are several edible tubers/stems such as taro (*Colocasia*), giant taro (*Alocasia*), tannia or yautia (*Xanthosoma*), elephant foot yam (*Amorphophallus*), and swamp taro (*Cyrtosperma*). The origin of tannia is South America and the Caribbean regions [4].* Colocasia*, originating in India and Southeast Asia, is a staple food in many islands of the South Pacific, such as Tonga and Western Samoa, and in Papua New Guinea. Furthermore, taro is the most widely cultivated crop in Asia, Africa, and Pacific as well as Caribbean Islands.

### 2.6. Minor Tuber Crops

#### 2.6.1. Canna

*Canna* is rhizomatous type tuber which is widely distributed throughout the tropics and subtropics. The genus* Canna* belongs to the family Cannaceae. The edible types of* Canna edulis* originated in the Andean region or Peruvian coast and extended from Venezuela to northern Chile, in South America. It is commercially cultivated in Australia for the production of starch.

#### 2.6.2. Arrowroot

*Maranta arundinacea* L. (West Indian arrowroot) is cultivated for its edible rhizomes. It belongs to* Marantaceae* and is believed to have originated in the Northwestern part of South America. Arrowroot has been widely distributed throughout the tropical countries like India, Sri Lanka, Indonesia, the Philippines, and Australia and West Indies.

### 2.7. Bioactive Compounds in Tuber Crops

Bioactive compounds in plants are secondary metabolites having pharmacological or toxicological effects in humans and animals. Secondary metabolites are produced within the plants besides the primary biosynthesis associated with growth and development. These compounds perform several essential functions in plants, including protection from undesirable effects, attraction of pollinators, or signaling of essential functions.

#### 2.7.1. Phenolic Compounds

Phenolic compounds have an aromatic ring with one or more hydroxyl groups and act as antioxidants. They are derived from biosynthetic precursors such as pyruvate, acetate, a few amino acids, acetyl-CoA, and malonyl-CoA following the pentose phosphate, shikimate, and phenylpropanoid metabolism pathways. In plants, phenylalanine and to a lesser extent tyrosine are the two major amino acids involved in the synthesis of phenolic compounds [3]. Major groups of phenolic compounds abundantly found in plants are simple phenolics, phenolic acids, flavonoids, coumarins, stilbenes, tannins, lignans, and lignins. The quantity of phenolic compounds present in a given species of plant material varies with a number of factors such as cultivar, environmental conditions, cultural practices, postharvest practices, processing conditions, and storage [3]. Two classes of phenolic acids, hydroxybenzoic acids and hydroxycinnamic acids, are found in plant materials. Compounds with a phenyl ring (C_6_) and a C_3_ side chain are known as phenylpropanoids and serve as precursors for the synthesis of other phenolic compounds. Flavonoids are synthesized by condensation of a phenylpropanoid compound with three molecules of malonyl coenzyme A. The phenolics present in tubers render several health benefits, namely, antibacterial, anti-inflammatory, and antimutagenic activities, among others.

#### 2.7.2. Saponins and Sapogenins

Saponins are high molecular weight glycosides consisting of a sugar moiety linked to a triterpene or steroid aglycone. The aglycone portion of the saponin molecule is called sapogenin. Depending on the type of sapogenin present saponins are divided into three groups, namely, triterpene glycosides, steroid glycosides, and steroid alkaloid glycosides. Saponins having a steroid structure are precursors for the chemical synthesis of birth control pills (with progesterone and estrogen), similar hormones, and corticosteroids [24]. According to recent findings steroidal saponins could be a novel class of prebiotics to lactic acid bacteria and are effective candidates for treating fungal and yeast infections in humans and animals [25].

#### 2.7.3. Bioactive Proteins

The protein contents of roots and tuber crops are variable. The global contribution of proteins from roots and tubers in the diet is less than 3%. However, in African countries, this contribution may vary from 5 to 15% [4].

Dioscorin is the main storage protein found in tropical* Dioscorea* yams. It accounts for 90% of water extractable soluble proteins in a majority of* Dioscorea* species. Dioscorin was reported to have carbonic anhydrase and trypsin inhibitor activities [26]. Furthermore, dehydroascorbate reductase and monodehydroascorbate reductase activities of dioscorin in the presence of glutathione have been reported [27]. Dioscorin from fresh yam (*Dioscorea batatas*) exhibited DPPH radical scavenging activity [28] and showed beneficial effects in lowering blood pressure [19, 29]. In addition, dioscorin demonstrated angiotensin converting enzyme (ACE) inhibitory and antihypertensive activities on spontaneously hypertensive rats [29, 30]. The dioscorin from yam exhibited carbonic anhydrase, trypsin inhibitor, dehydroascorbate reductase (DHA), and monodehydroascorbate reductase (MDA) activities and immunomodulatory activities [18, 26, 27].

Sporamin is a soluble protein and is the main storage protein in sweet potato roots and accounts for about 60–80% of its total proteins [31]. The sporamin of sweet potatoes is initially known as ipomoein. It is a nonglycoprotein without glycan and is stored in the vacuole in the monomeric form. Sporamin is initially produced as preprosporamin, which is synthesized by the membrane-bound polysome in the endoplasmic reticulum (ER) [32]. Sporamin is a trypsin inhibitor with a Kunitz-type trypsin inhibitory activity which has potential application in the transgenic insect resistant plants [33]. Furthermore, sporamin showed various antioxidant activities related to stress tolerance, such as DHA and MDA reductase activities [34].

#### 2.7.4. Glycoalkaloids

Glycoalkaloids are an important class of phytochemicals found in many species of the genera* Solanum* and* Veratrum* [35]. Alkaloids are nitrogen-containing secondary metabolites found mainly in several higher plants and microorganisms and animals [36]. The skeleton of most alkaloids is derived from amino acids and moieties from other pathways, such as those originating from terpenoids. The primary function of alkaloids in plants is acting as phytotoxins, antibactericides, insecticides, and fungicides and as feeding deterrents to insects, herbivorous mammals, and mollusks [37]. There are two main glycoalkaloids in commercial potatoes. These include *α*-chaconine and *α*-solanine which are glycosylated derivatives of the aglycone solonidine. Wild potatoes (*Solanum chacoense*) and egg plants contain the glycoalkaloid solasonine. The major glycoalkaloid reported in tomatoes is *α*-tomatine which is a glycosylated derivative of aglycone tomatidine. Steroidal alkaloids and their glycosides present in several species of* Solanum* are known to possess a variety of biological activities such as antitumour, antifungal, teratogenic, antiviral, and antiestrogenic activities. Certain glycoalkaloids are used as anticancer agents [38, 39]. The steroidal alkaloid glycosides showed cytotoxic activity against various tumour cell lines [40].

#### 2.7.5. Carotenoids

Carotenoids are among the most widespread natural pigments with yellow, orange, and red colors in plants. The carotenes are hydrocarbons soluble in nonpolar solvents such as hexane and petroleum ether. The oxygenated derivatives of carotenes, xanthophylls, dissolve better in polar solvents such as alcohols [41]. The majority of carotenoids are unsaturated tetraterpenes with the same basic C 40 isoprenoid skeleton resulting from the joining of eight isoprene units in a head-to-tail manner with the exception of the tail-to-tail connection at the centre. Carotenoids play important biological roles in living organisms. In photosynthetic systems of higher plants, algae, and phototrophic bacteria, carotenoids participate in a variety of photochemical reactions [42]. Carotenoids, either isolated from natural sources or chemically synthesized, have been widely utilized, due to their distinctive coloring properties, as natural nontoxic colorants in manufactured foods, drinks, and cosmetics. Carotenoids possess numerous bioactivities and play important roles in human health and nutrition, including provitamin A activity, antioxidant activity, regulation of gene expression, and induction of cell-to-cell communication [43], which are involved in a myriad of health beneficial effects. It has been demonstrated that zeaxanthin and lutein are stable throughout artificial digestion, whereas *β*-carotene and all-trans lycopene are degraded in the jejunal and ileal compartments. Among the isomers, the stability of 5-*cis* lycopene is superior to that of all-trans lycopene and 9-*cis* lycopene [44]. Yellow varieties of sweet potatoes and yams are good sources of carotenoids.

#### 2.7.6. Ascorbic Acid

Ascorbic acid, also known as vitamin C, is a water-soluble vitamin. It naturally occurs in plant tissues, primarily in fruits and vegetables. Ascorbic acid occurs in considerable quantities in several root crops. However, the level could be reduced during cooking of roots unless skins and cooking water are utilized. Root crops, if carefully prepared, can make a significant contribution to the vitamin C content of the diet. As reported by the 1983 Nutritional Food Survey Committee, potatoes serve as a principal source of vitamin C in British diets, providing 19.4% of the total requirement [2]. In general yams contain 6–10 mg of vitamin C/100 g and may vary up to 21 mg/100 g. In addition, the vitamin C content of potatoes is very similar to those of sweet potatoes and cassava. The concentration of ascorbic acid varies with the species, location, crop year, maturity at harvest, soil, and nitrogen and phosphate fertilizers [2].

## 3. Bioactivities of Phytochemicals in Roots and Tubers

### 3.1. Antioxidant Activity

Accumulating research evidences demonstrate that oxidative stress plays a major role in the development of several chronic diseases such as different types of cancer, cardiovascular diseases, arthritis, diabetes, autoimmune and neurodegenerative disorders, and aging. Though internal antioxidant defense systems, either enzymes (superoxide dismutase, catalase, and glutathione peroxidase) or other compounds (lipoic acid, uric acid, ascorbic acid, *α*-tocopherol, and glutathione), are available in the body, external sources of antioxidants are needed, as internal defense system may get overwhelmed by excessive exposure to oxidative stress. A number of studies have reported the antioxidant activities of several roots and tuber crops.

Methanolic extract of potatoes demonstrated high phenolic content and strong antioxidant activity as determined by 2,2-diphenyl-1-picrylhydrazyl (DPPH) radical scavenging activity [45]. Authors further showed that the total phenolic content (TPC) ranged from 16.6 to 32 mg gallic acid equivalents (GAE)/100 g dry sample and EC 50 of DPPH radical scavenging activity was 94 mg/mL (dry matter).

Several authors reported that the peels of sweet potato possessed a potent wound healing effect, which appears to be related to the free radical scavenging activity of the phytoconstituents and their ability in lipid oxidation inhibition [46, 47]. The healing effect of sweet potato fibre for burns or decubital wounds in a rat model was demonstrated and the reduction in size and changes in the quality of the wounds were observed [48]. They further found that the rats treated with sweet potato fibre covering had reduced wound area compared to those of the control. Petroleum ether extract of sweet potato had shown significant closure of scar area for complete epithelialization compared to the control [46].

The methanolic extracts of the peels and peel bandage of sweet potato roots were screened for wound healing effect by excision and incision wound models on Wistar rats [47]. They further showed that hydroxyproline content was found to be significantly increased in the test group compared to that of wounded control group. Increased hydroxyproline content leads to enhancement of collagen synthesis which improves wound healing. In addition, the content of malondialdehyde decreased in test groups compared to that of wounded control group, indicating lipid oxidation inhibitory effect of sweet potato peels [47]. Water yam (*Dioscorea alata*) was reported to possess the highest DPPH radical scavenging activity of 96% among different selected tuber crops such as sweet potato, potato, coco yam, and other* Dioscorea* yams (Table 3; [49]).

Cornago et al. [50] showed the TPC and antioxidant activities of two major Philippine yams of* Ube *(purple yam) and* Tugui* (lesser yam). Purple yam (*Dioscorea alata*) and lesser yam (*Dioscorea esculenta*) had a TPC which ranged from 69.9 to 421.8 mg GAE/100 g dry weight. The purple yam variety* Daking* had the highest TPC and antioxidant activities as measured by DPPH radical scavenging activity, reducing power and ferrous ion chelating capacity, whereas varieties* Sampero* and* Kimabajo* showed the lowest TPC and antioxidant activities.

Hsu et al. [51] studied the antioxidant activity of water and ethanolic extracts of yam peel on tert-butyl hydroperoxide (t-BHP) induced oxidative stress in mouse liver cells (Hepa 1–6 and FL83B). Ethanolic extracts of yam peel exhibited a better protective effect on t-BHP treated cells compared to that of water extracts. Furthermore, it was observed that ethanolic extract increased catalase activity, whereas water extract decreased it. According to Chen and Lin [52] heating affected the TPC, antioxidant capacity, and the stability of dioscorin of various yam tubers. Raw yams contained higher TPC than their cooked counterparts. Furthermore, the DPPH radical scavenging activities declined with increasing temperature. TPC and dioscorin content of yam cultivars (*Dioscorea alata* L. var. Tainung number 2) and keelung yam (*D. japonica* Thunb. var. pseudojaponica (Hay.) Yamam) correlated with DPPH radical scavenging activity and ferrous ion chelating effect [52]. Phytochemicals of yams seem to enhance the activities of endogenous antioxidant enzymes. The administration of yams decreased the levels of *γ*-glutamyl transpeptidase (GGT), low density lipoprotein, and triacylglycerol in serum of rats in which hepatic fibrosis was induced by carbon tetrachloride [53]. Treatment of rats with yams increased the antioxidant activities of hepatic enzymes, namely, glutathione peroxidase and superoxide dismutase [53].

Extracts of flavonoids and flavones from potatoes showed high scavenging activities toward oxygen radicals. Potatoes scavenged 94% of hydroxyl radicals [54]. The major phenolic compound reported in potato was chlorogenic acid which constituted more than 90% of its phenolics. The range of hydrophilic oxygen radical absorbance capacity (ORAC) of 200 to 1400 *μ*mol trolox equivalents/100 g fresh weight and the lipophilic ORAC range of 5 to 30 nmol alpha-tocopherol equivalents/100 g fresh weight were reported for potatoes [55].

Using a rat model, it was shown that ethanolic extracts of purple fleshed potato flakes had effective free radical scavenging activity and inhibition of linoleic acid oxidation [56]. Further potato extracts enhanced hepatic manganese superoxide dismutase (SOD), Cu/Zn-SOD, and glutathione peroxidase (GSH-Px) activities as well as mRNA expression, suggesting a reduced hepatic lipid peroxidation and an improved antioxidant potential.

Recently, Ji et al. [57] reported the contents of phenolic compounds and glycoalkaloids of 20 potato clones and their antioxidant, cholesterol uptake and neuroprotective activities* in vitro*. Peels of purple and red pigmented potato clones showed higher phenolic content compared to those of yellow and unpigmented clones [57]. Chlorogenic acid (50–70%) and anthocyanins, namely, pelargonidin and petunidin glycosides, were identified as major phenolic compounds present in potatoes. In addition, major glycoalkaloids present in potatoes *α*-chaconine and *α*-solanine and their contents were reduced by granulation process. Peels of potato clones showed the highest DPPH radical scavenging activity followed by flesh and granules [57].

A few studies have reported the antioxidant activities of cassava roots. A recent study [58] showed that the antioxidant activities of organically grown cassava tubers were higher than those of mineral-base fertilized roots. They found that TPC and flavonoid content (FC) were significantly higher for organic cassava compared to those of cassava grown with inorganic fertilizers.

### 3.2. Antiulcerative Activities

The antiulcerative activity of sweet potato roots was studied in a rat model [59]. The extract of sweet potatoes did not show any toxic or deleterious effects by oral route up to 2000 mg/kg. Furthermore, superoxide dismutase (SOD), catalase (CAT), glutathione peroxidase (GPx), and glutathione reductase (GR) activities were significantly elevated by administration of tuber extracts in treated rats, indicating the ability of restoring enzyme activities compared to the control. Kim et al. [60] showed that butanol fraction of sweet potato could be a better source for treating gastric ulcers induced by excessive alcohol intake.

### 3.3. Anticancer Activities

Cancer is a leading cause of death worldwide, and it is mostly related to unhealthy food habits and lifestyle. It is important to find ways to reduce and prevent the risk of cancer through dietary components, which are present in plant foods. Cancer is a multistage disease condition and tapping at any initial stage could help attenuate the disease condition. Root and tuber phytochemicals have demonstrated anticancer effects in several types of carcinoma cell lines and animal models.

Huang et al. [61] showed that aqueous extract of sweet potatoes had higher antiproliferative activity than that of ethanol extracts. Cell proliferation was analyzed at 48 h after human lymphoma NB4 cells which had been cultured with several concentrations of extracts, 0, 25, 50, 100, 200, 400, 800, or 1000 *μ*g of dry matter/mL, in the media using the microculture tetrazolium treatment (MTT) assay. The phytochemicals present in sweet potato roots may exert a significant effect on antioxidant and anticancer activities. Furthermore, antioxidant activity is directly related to the phenolics and flavonoid contents of the sweet potato extracts. An additive role of phytochemicals was also found, which may contribute significantly to the potent antioxidant activity and antiproliferative activity* in vitro* [61]. Furthermore, two anthocyanin pigments, namely, 3-(6,6′-caffeylferulylsophoroside)-5-glucoside of cyanidin (YGM-3) and peonidin (YGM-6), purified from purple sweet potatoes effectively inhibited the reverse mutation induced by mutagenic pyrolysates of tryptophan (Trp-P-1, Trp-P-2) and imidazoquinoline (IQ) in the presence of rat liver microsomal activation systems [62].

A greater inhibition of carcinogenesis was shown by red pigmented cultivar of potatoes compared to a white Russet Burbank cultivar in breast cancer induced rats [63]. The red cultivar of potatoes was reported to have high levels of anthocyanins and chlorogenic acid derivatives.

According to Madiwale et al. [64], purple fleshed potato showed higher potential in suppressing proliferation and elevated apoptosis of HT-29 human colon cancer cell lines compared with white fleshed potato. It was interesting to note that storage of potatoes affected their antioxidant and anticancer activities and TPC. The extracts from both fresh and stored potatoes inhibited cancer cell proliferation and elevated apoptosis, but anticancer effects were higher in fresh potatoes than in stored tubers. The study further demonstrated that storage duration had a strong positive correlation with antioxidant activity and percentage of viable cancer cells and a negative correlation existed with apoptosis induction. These findings further elaborated that antioxidant activity and phenolic content of potatoes increased with storage, but antiproliferative and proapoptotic activities were decreased [64].

In addition to phenolic compounds, saponins present in roots and tubers play a pivotal role as anticancer/antitumour agents. There are several groups of saponins, namely, cycloartanes, ammaranes, oleananes, lupanes, and steroids, demonstrating strong antitumour effect on different types of cancers. For instance, cycloartane saponins possess antitumour properties in human colon cancer cells and tumour xenografts [65]. They downregulated expression of the hepatocellular carcinoma (HCC) tumour marker *α*-fetoprotein and suppressed HepG2 cell growth by inducing apoptosis and modulating an extracellular signal-regulated protein kinase- (ERK-) independent NF-*κ*B signaling pathway [65]. Furthermore, oleananes saponins exerted their antitumour effect through various pathways, such as anticancer, antimetastasis, immunostimulation, and chemoprevention pathways [65].

Several studies have shown that glycoalkaloids such as *α*-chaconine and *α*-solanine present in tubers are potential anticarcinogenic agents [66]. Glycoalkaloids showed antiproliferative activities against human colon (HT-29) and liver (HepG2) cancer cells as assessed by the MTT assay [66].

Wang et al. [67] reported that the aqueous extract of yam (*Dioscorea alata*) inhibited the H_2_O_2_-CuSO_4_ induced damage of calf thymus DNA and protected human lymphoblastoid cells from CuSO_4_ induced DNA damage. Extract of water yam contains a homogenous compound with a single copper-binding site and also is a good natural, safe (redox inactive) copper chelator. In addition to phenolic compounds saponins and mucilage polysaccharides present in yams are responsible for this activity. Furthermore, water-soluble mucilage polysaccharides are the most important copper chelators in extract of water yam. Thus* Dioscorea alata* aqueous extracts could serve as potential agents in the management of copper-mediated oxidative disorders and diabetes [67].

### 3.4. Immunomodulatory Activities

Purified dioscorin from yam tubers showed immunomodulatory activities* in vitro* [18]. The effects of dioscorin on native BALB/c mice spleen cell proliferation were assayed by MTT assay. It was found that dioscorin stimulated RAW 264.7 cells to produce nitric oxide (NO), in the absence of lipopolysaccharide (LPS) contamination. Yam dioscorin exhibited immunomodulatory activities by the innate immunity which is a nonspecific immune system which comprises the cells and mechanisms that defend the host from infection by other organisms in a nonspecific manner. Dioscorin was reported to stimulate cytokine production and to enhance phagocytosis. Furthermore, the released cytokines may act synergistically with phytohemagglutinin (PHA) which is a lectin found in plants that stimulate the proliferation of splenocytes [18].

Several studies have demonstrated the immune activity of yam mucopolysaccharides (YMP).* In vitro* cytotoxic activity of mouse splenocyte against leukemia cell was increased in the presence of YMP of* Dioscorea batatas* at 10 *μ*g/mL [68]. Furthermore, the production of IFN-*γ* was significantly increased in the YMP treated splenocytes, suggesting their capability of inducing cell-mediated immune responses. In addition, YMP at a concentration of 50 *μ*g/mL increased uptaking capacity and lysosomal phosphatase activity of peritoneal macrophages [68].

*Dioscorea *phytocompounds enhanced murine splenocyte proliferation* ex vivo* and improved regeneration of bone marrow cells* in vivo* [69]. Mice which were fed with a* Dioscorea *extract recovered damaged bone marrow progenitor cell populations that had been depleted by large doses of 5-fluorouracil (5-FU). Furthermore, they reported that the phytocompound(s) responsible for these bioactivities had a high molecular weight (*≥*100 kDa) and were most likely polysaccharides. They postulated those high-molecular-weight polysaccharides in DsCE-II act on specific target cell types in the GI tract (dendritic cells, intestinal epithelial cells, and T-cells) to mediate a cascade of immunoregulatory activities leading to the recovery of damaged cell populations following 5-FU or other chemical insults in the bone marrow, spleen, or other immune cell systems [69].

Dioscorea tuber mucilage from Taiwanese yams (*Dioscorea Japonica* Thunb var.) showed significant effects on the innate immunity and adaptive immunity on BALB/c mice through oral administration [70]. In addition, it was found that the specific antibodies rapidly responded against foreign proteins (or antigens) in the presence of yam mucilage. Mucilage from these yam varieties exhibited a stimulatory effect on phagocytic activity by granulocyte and monocyte (*ex vivo*), on peritoneal macrophages, and on the RAW 264.7 cells (*in vivo*) of mice [70].

Yams (*Dioscorea esculenta*) showed anti-inflammatory activity on carrageenan induced oedema in the right hind paw of Wistar rats [71]. However, this activity was short lived as it was quickly removed from the system after reaching the peak within 2 hours. Phytochemical screening of* D*.* esculenta* confirmed the presence of saponins, *β*-sitosterol, stigmasterol, cardiac glycosides, fats, starch, and diosgenin, which could be responsible for the observed activity [71]. Diosgenin contained in Chinese yam was an immunoactive steroidal saponin which also showed prebiotic effect. Diosgenin had also beneficial effects on the growth of enteric lactic acid bacteria [25].

The impact of the consumption of pigmented potatoes on oxidative stress and inflammatory damages has been demonstrated in humans [72]. Participants were given white, yellow (high concentrations in phenolic acids and carotenoids), or purple fleshed (high content of anthocyanin and phenolic acids) potato once per day in a randomized 6-week trial which reported good compliance. The results showed that the consumption of pigmented potatoes was responsible for elevated antioxidant status and reduced inflammation and DNA damage, which was observed through the reduction of inflammatory cytokine and C-reactive protein concentrations [72]. Methanolic extracts of* A. campanulatus* (Suran) tuber also showed immunomodulatory activity [73]. Authors indicated that the presence of steroids and flavonoids in* A. campanulatus* (Suran) tuber may be responsible for the observed immunomodulatory activity [73].

### 3.5. Antimicrobial Activity

Yam varieties with their phenolic compounds are potential agents with antimicrobial efficacy. Sonibare and Abegunde [74] reported that the methanolic extracts of* Dioscorea *yams (*Dioscorea dumetorum* and* Dioscorea hirtiflora*) showed antioxidant and antimicrobial activities. Antimicrobial activity was determined by the agar diffusion method (for bacteria) and pour plate method (for fungi). Nonedible* D*.* dumetorum* showed the highest* in vitro* antibacterial activity against* Proteus mirabilis*. The methanolic extracts from* D*.* hirtiflora* demonstrated antimicrobial activity against all tested organisms, namely,* Staphylococcus aureus*,* E coli*,* Bacillus subtilis, Proteus mirabilis, Salmonella typhi, Candida albicans, Aspergillus niger*, and* Penicillium chrysogenum*.

### 3.6. Hypoglycemic Activities

Diabetes mellitus is a chronic disorder marked by elevated levels of glucose in the blood and life-threatening complications that can untimely lead to death. Extracts of sweet potato peels have shown reduced plasma glucose levels of diabetic patients [75]. The extract of white skinned sweet potatoes (WSSP) reduced hyperinsulinemia in Zucker fatty rats by 23, 26, 60, and 50%, after 3, 4, 6, and 8 weeks, respectively, starting the oral administration of WSSP similar to troglitazone (insulin sensitizer) [76]. In addition, blood triacylglycerol (TG), free fatty acid (FFA), and lactate levels were also lowered by the oral administration of WSSP. In histological examinations of the pancreas of Zucker fatty rats, remarkable regranulation of pancreatic islet B-cells was observed in the WSSP and troglitazone groups after 8 weeks of treatment. Based on these findings it was concluded that WSSP was likely to improve the abnormal glucose and lipid metabolism in insulin-resistant diabetes mellitus. In support of these observations, ingestion of 4 g of caiapo, the extract of WSSP, per day for 6 weeks reduced fasting blood glucose and total as well as low density lipoprotein (LDL) cholesterol in male Caucasian type 2 diabetic patients who were previously treated by diet alone [75]. However, significant changes were not observed between low dose of caiapo and placebo. The improvement of insulin sensitivity, as determined by the frequently sampled intravenous glucose tolerance test (FSIGT), indicated that caiapo extracts showed beneficial effects via reducing insulin resistance. Later Ludvik et al. [77] confirmed the beneficial effects of caiapo on glucose and serum cholesterol levels in type 2 diabetic patients treated by diet alone for 3 months after administration for the study. Improved fasting blood glucose levels and glucose levels during an OGTT and in the postprandial state as well as improvement in long-term glucose control were also observed as expressed by the significant decrease in HbA1c [77].

Ethanolic extract of tubers of* Dioscorea alata* showed an antidiabetic activity in alloxan-induced diabetic rats [78]. Diabetic rats with administered yam extract exhibited significantly lower creatinine levels which could be a result of improved renal function by reduced plasma glucose level and subsequent glycosylation of renal basement membranes. Several bioactive compounds, including phenolics, were identified in the ethanolic extract of* D. alata*. These include hydro-Q9 chromene, *γ*-tocopherol, *α*-tocopherol, feruloyl glycerol, dioscorin, cyanidin-3-glucoside, catechin, procyanidin, cyanidin, peonidin 3-gentiobioside, and alatanins A, B, and C [78].

### 3.7. Hypocholesterolemic Activity

Cardiovascular diseases are among the leading causes of death worldwide. It is well known that diet plays an important role in the regulation of cholesterol homeostasis. External agents possessing anticholesterolemic activities continuously show beneficial effects on risk reduction and management of the disease conditions.

Diosgenin, a steroidal saponin of yam (*Dioscorea*), demonstrated antioxidative and hypolipidemic effects* in vivo* [79]. Rats fed with a high-cholesterol diet were supplemented with either 0.1 or 0.5% diosgenin for 6 weeks. The lipid profile of the plasma and liver, lipid peroxidation and antioxidant enzyme activities in the plasma, erythrocyte, and gene expression of antioxidative enzymes in the liver and the oxidative DNA damage in lymphocytes were measured. Diosgenin showed pancreatic lipase inhibitory activity, protective effect of liver under high-cholesterol diet, reduced total cholesterol level, and protection against the oxidative damaging effects of polyunsaturated fatty acids [79]. Steroidal saponins of yams are used for industrial drug processing. Saponins, such as dioscin and gracilin, and prosapogenins of dioscin have long been identified from yam. The content of dioscin was about 2.7% (w/w). Diosgenin content was about 0.004 and 0.12–0.48% in cultivated yam and wild yam, respectively. The antihypercholesterolemic effect of yam saponin is related to its inhibitory activity against cholesterol absorption [80].

The effect of yam diosgenin on hypercholesterolemia had been also reported by Cayen and Dvornik [81]. Hypercholesterolemic rats fed with yam (*Dioscorea*) showed that diosgenin had decreased cholesterol absorption, increased hepatic cholesterol synthesis, and increased biliary cholesterol secretion without affecting serum cholesterol level. In agreement with this finding, several studies showed that diosgenin, in some* Dioscorea*, could enhance fecal bile acid secretion and decrease intestinal cholesterol absorption [82, 83]. The relative contribution of biliary secretion and intestinal absorption of cholesterol in diosgenin-stimulated fecal cholesterol excretion were studied using wild-type (WT) and Niemann-Pick C1-Like 1 (NPC1L1) knockout (LIKO) mice. NPC1L1 was recently identified as an essential protein for intestinal cholesterol absorption [84]. Diosgenin significantly increased biliary cholesterol and hepatic expression of cholesterol synthetic genes in both WT and LIKO mice. In addition diosgenin stimulation of fecal cholesterol excretion was primarily attributable to its impact on hepatic cholesterol metabolism rather than NPC1L1-dependent intestinal cholesterol absorption [85].

Native protein of dioscorin purified from* D. alata* (cv. Tainong number 1) (TN1-dioscorin) and its peptic hydrolysates presented ACE inhibitory activities in a dose-dependent manner [29]. According to kinetic analysis, dioscorin showed mixed noncompetitive inhibition against ACE. Dioscorin from* Dioscorea* might be beneficial in controlling high blood pressure [85].

Chen et al. [23] reported the effects of Taiwan's yam (*Dioscorea alata* cv. Tainung number 2) on mucosal hydrolase activities and lipid metabolism in male Balb/c mice. High level of Tainung number 2 yam in the diet (50% w/w) reduced plasma and hepatic cholesterol levels and increased fecal steroid excretions in mice model. This could be due to the loss of bile acid in the enterohepatic cycle to fecal excretion [23]. They further suggested that the increased viscosity of the digest and the thickness of the unstirred layer in the small intestine caused by Tainung number 2 yam fibre (or/and mucilage) decreased the absorption of fat, cholesterol, and bile acid. Short term (3-week) consumption of 25% Tainung number 2 yam in the diet could reduce the atherogenic index but not total cholesterol level in nonhypercholesterolemic mice. Furthermore, additional dietary yam (50% yam diet) could consistently exert hypocholesterolemic effects in these mice [23]. However, Diosgenin was not elucidated in Tainung number 2 used in this study [23]. Thus, authors suggested that diosgenin might not be involved in the cholesterol lowering effect of Tainung number 2 yam. Dietary fibre and viscous mucilage could be active components for the beneficial cholesterol lowering effects of yam. Furthermore, short term consumption (3-week) of 25% uncooked keelung yam effectively reduced total blood cholesterol levels and the atherogenic index in mice. Authors indicated that the active components for the lipid lowering effects may be attributed to dietary fibre, mucilage, plant sterols, or synergism of these active components [23].

### 3.8. Hormonal Activities

Yam (*Dioscorea*) has the ability to reduce the risk of cancer and cardiovascular diseases in postmenopausal women [86]. It was shown that the levels of serum estrogen and sex hormone binding globulin (SHBG) increased significantly after subjects had been on a yam diet for 30 days. Furthermore, three serum hormone parameters measured, namely, estrogen, estradiol, and SHBG, did not change in those who were fed with sweet potatoes as the control. The risk of breast cancer which increased by estrogens might be balanced by the elevated SHB and the ratio of estrogen plus estradiol to SHBG. Authors further showed that high SHBG levels had a protective effect against the occurrence of type 2 diabetes mellitus and coronary heart diseases in women [86].

Chronic administration of* Dioscorea* may enhance bone strength and provide insight into the role of* Dioscorea* in bone remodeling and osteoporosis during the menopause [87]. Administration of* Dioscorea* to ovariectomised rats decreased the porosity effect on bones and increased the ultimate force of bones. The changes in biochemical and physiological functions seen in these animals were similar to those in menopausal women [87].

### 3.9. Antiobesity

Differentiation and proliferation inhibitory activity of sporamin of sweet potato roots in 3T3-L1 preadipocytes were reported [84]. Sporamin did not exhibit any cytotoxic activity toward the model cell line 3T3-L1 preadipocytes which have frequently been used to study differentiation of adipocytes* in vitro*. Concentration range of 0.025 to 1 mg/mL sporamin showed antidifferentiation and antiproliferation effects on 3T3-L1 cells similarly to 0.02 mg/mL berberine. Berberine is a traditional Chinese medicine used as an antimicrobial and antitumour agent [84]. Hwang et al. [88] demonstrated that purple sweet potato is a potential agent, which can be used for the prevention of obesity. Anthocyanin fractions of purple sweet potato inhibited hepatic lipid accumulation through the induction of adenosine monophosphate activated protein kinase (AMPK) signaling pathways. AMPK plays an important role in the regulation of lipid synthesis in metabolic tissues [88]. Anthocyanin dose of 200 mg/kg of body weight per day reduced weight gain and hepatic triacylglycerol accumulation and improved serum lipid parameters in mice fed for 4 weeks with purple sweet potatoes. Furthermore, anthocyanin administration increased the phosphorylation of AMPK and acetyl coenzyme A carboxylase (ACC) in the liver and HepG2 hepatocytes. These authors further suggested that anthocyanin fraction may improve high fat diet induced fatty liver disease and regulate hepatic lipid metabolism [88].

## 4. Effect of Processing on Bioactivities of Roots and Tubers

Tuber crops are processed in a number of ways before they are consumed and these include hydrothermal treatments such as boiling, frying, baking and roasting, dehydration, and fermentation. Depending on the region and country different types of foods are prepared at domestic and industrial levels.

Major proportions of cassava produced in Africa and Latin America are processed into fermented foods and food additives such as organic acids and monosodium glutamate. Lactic acid bacteria and yeasts are two major groups of microorganisms used for cassava fermentation. Those fermented foods from cassava include* gari*,* fufu*,* lafun*,* chickwanghe*,* agbelima*,* attieke*, and* kivunde* in Africa, tape in Asia, and “cheese” bread and “coated peanut” in Latin America [89]. Sweet potatoes also may be fermented into soy sauce, vinegar, lactojuices, lactopickles, and* sochu* (an alcoholic drink produced in Japan), and yams may be fermented into fermented flour. Furthermore, yams are used in a number of foods. Greater yams (*Dioscorea alata* L.), commonly known as* ube* in the Philippines, are utilized in sweetened food delicacies due to their attractive violet color and unique taste.* Ube *is used in a number of native delicacies such as* halaya* (yam pudding with milk),* sagobe* (with parboiled chocomilk and glutinous rice balls),* puto* (rice cake),* halo-halo*,* hopia*, and different types of rice cake using glutinous rice such as* suman*,* sapin-sapin*,* bitso*, and* bibingka*. It can also be used as an ingredient for flavour and/or filling in ice cream, tarts, bread, and cakes. Different food processing conditions alter the content of phytochemicals as well as their bioactivities.

Processing conditions, such as peeling, drying, and sulphite treatment, could change physiochemical properties and nutritional quality of sweet potato flour [90]. Sweet potato flour is generally used to enhance characteristics of food products through color, flavour, and natural sweetness and is supplemented nutrients. It also serves as substitute for cereal flours, which particularly contain gluten which is not suited for individuals diagnosed with celiac disease [91]. Peeled and unpeeled sweet potato flour with or without sulphite treatment showed higher browning indices at 55°C and decreased with increasing drying temperatures [90]. Furthermore, *β*-carotene content of all flours decreased with increasing temperature. Total phenolic content decreased at a higher drying temperature for peeled and unpeeled sweet potato flour without sulphite treatment. Unpeeled sweet potato flour had higher phenolic content which was contributed by the tuber skin [92]. Sweet potato flour with sulphite treatment showed higher phenolics and ascorbic acid contents and this could be due to the inactivation of polyphenol oxidase by sulphites. Sulphite reacts with quinines and inhibits the polyphenol oxidase activity and depletes oxygen content [93].

Shih et al. [94] demonstrated the physiochemical and physiological properties and bioactivities of sweet potato dry products made from two varieties, yellow (variety: Tainong 57) and orange (variety: Tainong 66), using different processing methods such as freeze-drying, hot air-drying, and extrusion. Antioxidant contents were different between 70% methanolic extracts of yellow and orange sweet potatoes. In addition, differences of antioxidant activity were observed among freeze-dried, hot air-dried, and extruded samples [94]. The freeze-dried samples of orange potatoes showed high phenolics, *β*-carotene and anthocyanin contents, and free radical scavenging activities compared to those of yellow sweet potatoes. The extrusion process significantly increased the DPPH radical scavenging activity and TPC, whereas anthocyanin and *β*-carotene contents were decreased. The increase in the level of phenolic acids after extrusion could be due to the release of the bound phenolic acids and their derivatives from the cell walls of the plant matter [94]. The DPPH radical scavenging activity was higher for hot air-dried samples compared to those of freeze-dried yellow sweet potatoes but this trend was opposite for orange sweet potatoes [94]. They also reported that the methanolic extracts of sweet potatoes had immunomodulatory activities. Methanolic extracts of sweet potatoes increased the proliferation of the lectin concanavalin A (Con A) stimulated splenocytes of BALB/c mice in a concentration dependent manner. Freeze-dried sweet potatoes had the highest mitogenic index compared to that of hot air-drying and extrusion processing samples of both yellow and orange sweet potatoes. Phenolic compounds, anthocyanins, and *β*-carotene could be responsible compounds for the observed bioactivities [94, 95].

The retention of *β*-carotene decreased with the duration of boiling, steaming, and microwave cooking [96]. It was found that 50-minute boiling reduced one-third of *β*-carotene content. Furthermore, steaming reduced higher content of *β*-carotene compared to that of boiling. It was noted that microwave cooking had the highest loss. Authors suggested that long cooking time leads to high reductions in *β*-carotene content of sweet potatoes [96].

Different cooking methods affect the phenolic contents of potatoes [97]. A significant loss in phenolic contents of peeled and unpeeled potatoes was observed during boiling and baking. Furthermore, boiling caused loss of protocatechuic and caffeoylquinic acids of peeled potatoes by 86 and 26%, respectively. In addition, microwave cooking resulted in loss of 50 to 83% of protocatechuic acid and 27 to 64% of caffeoylquinic acids in peeled potatoes. Interestingly the losses were reported to be lower in unpeeled potatoes than those in peeled potatoes [97]. The reported losses in phenolic contents could be due to a combination of degradation caused by leaching into water, heat, and polyphenol oxidation [10, 98]. Potatoes cooked with peel had a high amount of total phenolics in the cortex and internal tissues [99]. This has been attributed to the migration of phenolics from the peel into both cortex and internal tissues of the tuber [10]. Navarre et al. [100] also showed the effects of cooking (microwave cooking, boiling, and steaming) on the phenolic contents of potatoes and the results showed that none of these methods decreased the content of chlorogenic, cryptochlorogenic, and neochlorogenic acids. Furthermore, it was found that flavonols (rutin) remained during cooking; however, there was an increase in phenolic contents of stir-fried potatoes. Different processing methods decreased the anthocyanin content due to the effect of high temperature, enzyme activity, change in pH, and presence of metallic ions and proteins [101, 102]. Another study showed that anthocyanin content in raw potatoes was higher than that of potato chips and French fries which were processed by deep frying [103].

Fang et al. [103] identified the major phenolic compounds in Chinese purple yam and their changes during vacuum frying (Table 4). They demonstrated that 40 and 63% of anthocyanin remained after blanching and vacuum frying, respectively [103]. Furthermore, hydroxycinnamic acids, namely, sinapic acids and ferulic acids, showed higher stability than that of anthocyanins and freezing did not influence the phenolic contents due to short freezing time of 20 hours [103].

Different dehydration methods used to produce yam flours could affect their antioxidant activities [104]. Freeze-dried yam flours had the strongest DPPH radical scavenging activities compared to tjose of hot air-dried or drum-dried yam flours. Chen and Lin [52] also reported the negative impact of temperature on the content of phenolic compounds and dioscorin and antioxidant activities of yam cultivars. Chen et al. [104] further showed the effect of pH on phenolic contents, antioxidant activity, and stability of dioscorin in various yam tubers. The TPC observed of yam varieties were the highest at pH 5 and gradual decrease was observed with increased pH. The DPPH radical scavenging activity showed a similar pattern to those of phenolic contents of yams at pH 5, but ferrous ion chelating capacity was found to be high for all yams at pH 8 [104].

## 5. Conclusions

Roots and tubers are important diet components for humans and add variety to it. In addition to the main role as an energy contributor, they provide a number of desirable nutritional and health benefits such as antioxidative, hypoglycemic, hypocholesterolemic, antimicrobial, and immunomodulatory activities. A variety of foods can be prepared using tubers and type and usage vary with the country and region. Processing affects the bioactivities of constituent compounds. Tubers may serve as functional foods and nutraceutical ingredients to attenuate noncommunicable chronic diseases and to maintain wellness.

## Figures and Tables

**Table 1:** Nutritional composition of selected tuber crops.

Nutrients (per 100 g)	Potatoes		Sweet potatoes, raw	Cassava, raw	Yam, raw
	White flesh and skin, raw	Red flesh and skin, raw			
Proximate composition					
Energy (kcal)	69.0	70	86.0	160.0	118.0
Protein (g)	1.7	1.9	1.6	1.4	1.5
Total lipid (fat) (g)	0.1	0.1	0.1	0.3	0.2
Carbohydrate, by difference (g)	15.7	15.9	20.1	38.1	27.9
Fibre, total dietary (g)	2.4	1.7	3.0	1.8	4.1
Sugars, total (g)g	1.2	1.3	4.2	1.7	0.5
Minerals					
Calcium, Ca (mg)	9	10	30	16	17
Magnesium, Mg (mg)	21	22	25	21	21
Potassium, K (mg)	407	455	337	271	816
Phosphorus, P (mg)	62	61	47	27	55
Sodium, Na (mg)m	16	18	55	14	9
Vitamins					
Total ascorbic acid (mg)	19.70	8.60	2.40	20.60	17.10
Thiamin (mg)	0.07	0.08	0.08	0.09	0.11
Riboflavin (mg)	0.03	0.03	0.06	0.05	0.03
Niacin (mg)	1.07	1.15	0.56	0.85	0.55
Vitamin B-6 (mg)	0.203	0.170	0.209	0.088	0.293
Folate (*μ*g-DFE)	18	18	11	27	23
Vitamin E (mg)	0.01	0.01	0.26	0.19	0.35
Vitamin K (*μ*g)	1.6	2.9	1.8	1.9	2.3
Vitamin A (IU)IU	8	7	14187	13	138

Source: USDA [105].

**Table 2:** Different types of tuber crops commonly consumed in world.

	Botanical name	Family	Common name
Potatoes	*Solanum tuberosum*	Solanaceae	

Country potato Hausa potato	*Solenostemon rotundifolius *	Lamiaceae (mint family)	Innala, ratala (Sri Lanka)

Cannas	*Canna edulis*	Cannaceae	Buthsarana (Sri Lanka)

	*Maranta arundinacea *L.	Marantaceae	Arrow root Hulankeeriya (Sri Lanka) Aru aru, arawak (India)

Taro	*Xanthosoma sagittifolium*	Araceae	Kiriala (Sri Lanka) Keladi (Malaysia) Phueak (Thailand) Khoai mon (Vietnam) Sato-imo (Japan)

Yam	*Dioscorea alata*	Dioscoreaceae	Purple yam; greater yam Guyana; water yam Winged yam Raja ala (Sri Lanka) Ube (Philippines)

Sweet potatoes	*Ipomoea batatas*	Convolvulaceae	Camote; batata Shakarkand

Cassava	*Manihot esculenta*	Euphorbiaceae	Yuxco; mogo; manioc mandioca; kamoteng kahoy

Elephant foot yam	*Amorphophallus paeoniifolius*	Araceae	White pot giant arum; stink lily

**Table 3:** Percentage of 2,2-diphenyl 1-1-picrylhydrazyl (DPPH) radical scavenging activity, flavonoids, and phenolic content of selected tuber crops.

	% DPPH inhibition	Flavonoids	Total phenolics
St. Vincent yam (*Dioscorea alata*)	18.9 ± 0.56	390.65 ± 40.63	16.03 ± 0.79
Water yam (*Dioscorea alata*)	95.83 ± 0.21	410.52 ± 20.22	13.10 ± 1.03
Coco yam (*Xanthosoma *sp.)	12.59 ± 0.66	145.31 ± 5.61	9.39 ± 0.68
Sweet potatoes (*Ipomoea batatas*)	28.01 ± 1.34	165.34 ± 5.81	4.37 ± 0.27
Potatoes (*Solanum tuberosum*)	20.47 ± 1.38	85.21 ± 4.32	18.26 ± 1.35
Yellow yam (*Dioscorea cayenensis*)	13.55 ± 0.52	150.67 ± 30.34	3.43 ± 0.19

Source: Dilworth et al. [49].

**Table 4:** Content of phenolic compounds during various processing methods of purple yam (*Dioscorea alata*).

	Fresh yam	Blanched	Frozen	Vacuum frying
Total phenolic content (mg gallic acid equivalent/100 g)	478 ± 11.62	306 ± 7.7	293 ± 8.93	204 ± 5.49
Total anthocyanin content (mg/100 g)	31.0 ± 2.35	12.6 ± 1.65	12.6 ± 1.54	7.92 ± 1.03
Sinapic acid (mg/100 g)	135 ± 5.27	93.1 ± 7.53	91.73 ± 5.71	55.5 ± 3.32
Ferulic acid (mg/100 g)	31.3 ± 1.08	15.94 ± 1.15	15.77 ± 1.05	9.48 ± 1.11

Source: Fang et al. [103].

## References

[1] FAOSTAT http://faostat3.fao.org.

[2] Food and Agriculture Organization (FAO) (1990). *Roots, Tubers, Plantains and Bananas in Human Nutrition*.

[3] Naczk M., Shahidi F. (2006). Phenolics in cereals, fruits and vegetables: occurrence, extraction and analysis. *Journal of Pharmaceutical and Biomedical Analysis*.

[4] FAO (1999). *Production Year Book*.

[5] Burlingame B., Mouillé B., Charrondière R. (2009). Nutrients, bioactive non-nutrients and anti-nutrients in potatoes. *Journal of Food Composition and Analysis*.

[6] Hale A. L., Reddivari L., Nzaramba M. N., Bamberg J. B., Miller J. C. (2008). Interspecific variability for antioxidant activity and phenolic content among *Solanum* species. *American Journal of Potato Research*.

[7] Navarre D. A., Goyer A., Shakya R., Singh J., Kaur L. (2009). Nutritional value of potatoes; Vitamin, phyto-nutrient and mineral content. *Advances in Potatoes Chemistry and Technology*.

[8] McNulty H., Pentieva K. (2004). Folate bioavailability. *Proceedings of the Nutrition Society*.

[9] Diretto G., Al-Babili S., Tavazza R., Papacchioli V., Beyer P., Giuliano G. (2007). Metabolic engineering of potato carotenoid content through tuber-specific overexpression of a bacterial mini-pathway. *PLoS ONE*.

[10] Ezekiel R., Singh N., Sharma S., Kaur A. (2013). Beneficial phytochemicals in potato-a review. *Food Research International*.

[11] Scott G. J. (1992). Transforming traditional food crops: product development for roots and tubers. *Product Development for Root and Tuber Crops*.

[12] Bovell-Benjamin A. C. (2007). Sweet potato: a review of its past, present, and future role in human nutrition. *Advances in Food and Nutrition Research*.

[13] van Jaarsveld P. J., Marais D. W., Harmse E., Nestel P., Rodriguez-Amaya D. B. (2006). Retention of *β*-carotene in boiled, mashed orange-fleshed sweet potato. *Journal of Food Composition and Analysis*.

[14] Nassar N. M. A., Hashimoto D. Y. C., Fernandes S. D. C. (2008). Wild Manihot species: botanical aspects, geographic distribution and economic value. *Genetics and Molecular Research*.

[15] Blagbrough I. S., Bayoumi S. A. L., Rowan M. G., Beeching J. R. (2010). Cassava: an appraisal of its phytochemistry and its biotechnological prospects—review. *Phytochemistry*.

[16] Prawat H., Mahidol C., Ruchirawat S. (1995). Cyanogenic and non-cyanogenic glycosides from *Manihot esculenta*. *Phytochemistry*.

[17] Reilly K., Gómez-Vásquez R., Buschmann H., Tohme J., Beeching J. R. (2004). Oxidative stress responses during cassava post-harvest physiological deterioration. *Plant Molecular Biology*.

[18] Liu Y.-W., Shang H.-F., Wang C.-K., Hsu F.-L., Hou W.-C. (2007). Immunomodulatory activity of dioscorin, the storage protein of yam (*Dioscorea alata* cv. Tainong No. 1) tuber. *Food and Chemical Toxicology*.

[19] Iwu M. M., Okunji C. O., Ohiaeri G. O., Akah P., Corley D., Tempesta M. S. (1990). Hypoglycaemic activity of dioscoretine from tubers of *Dioscorea dumetorum* in normal and alloxan diabetic rabbits. *Planta Medica*.

[20] Bhandari M. R., Kasai T., Kawabata J. (2003). Nutritional evaluation of wild yam (*Dioscorea* spp.) tubers of Nepal. *Food Chemistry*.

[21] Kelmanson J. E., Jäger A. K., van Staden J. (2000). Zulu medicinal plants with antibacterial activity. *Journal of Ethnopharmacology*.

[22] Chan Y.-C., Hsu C.-K., Wang M.-F., Su T.-Y. (2004). A diet containing yam reduces the cognitive deterioration and brain lipid peroxidation in mice with senescence accelerated. *International Journal of Food Science and Technology*.

[23] Chen H.-L., Wang C.-H., Chang C.-T., Wang T.-C. (2003). Effects of Taiwanese yam (*Dioscorea japonica* Thunb var. pseudojaponica Yamamoto) on upper gut function and lipid metabolism in Balb/c mice. *Nutrition*.

[24] Podolak I., Galanty A., Sobolewska D. (2010). Saponins as cytotoxic agents: a review. *Phytochemistry Reviews*.

[25] Huang C.-H., Cheng J.-Y., Deng M.-C., Chou C.-H., Jan T.-R. (2012). Prebiotic effect of diosgenin, an immunoactive steroidal sapogenin of the Chinese yam. *Food Chemistry*.

[26] Hou W.-C., Chen H.-J., Lin Y.-H. (1999). Dioscorins, the major tuber storage proteins of yam (*Dioscorea batatas* Decne), with dehydroascorbate reductase and monodehydroascorbate reductase activities. *Plant Science*.

[27] Hou W.-C., Chen H.-J., Lin Y.-H. (2000). Dioscorins from different *Dioscorea* species all exhibit both carbonic anhydrase and trypsin inhibitor activities. *Botanical Bulletin of Academia Sinica*.

[28] Hou W.-C., Lee M.-H., Chen H.-J. (2001). Antioxidant activities of dioscorin, the storage protein of yam (*Dioscorea batatas* Decne) tuber. *Journal of Agricultural and Food Chemistry*.

[29] Hsu F.-L., Lin Y.-H., Lee M.-H., Lin C.-L., Hou W.-C. (2002). Both dioscorin, the tuber storage protein of yam (*Dioscorea alata* cv. Tainong No. 1), and its peptic hydrolysates exhibited angiotensin converting enzyme inhibitory activities. *Journal of Agricultural and Food Chemistry*.

[30] Lin J.-Y., Lu S., Liou Y.-L., Liou H.-L. (2006). Antioxidant and hypolipidaemic effects of a novel yam-boxthorn noodle in an in vivo murine model. *Food Chemistry*.

[31] Shewry P. R. (2003). Tuber storage proteins. *Annals of Botany*.

[32] Senthilkumar R., Yeh K.-W. (2012). Multiple biological functions of sporamin related to stress tolerance in sweet potato (*Ipomoea batatas* Lam). *Biotechnology Advances*.

[33] Yeh K.-W., Chen J.-C., Lin M.-I., Chen Y.-M., Lin C.-Y. (1997). Functional activity of sporamin from sweet potato (*Ipomoea batatas* Lam.): a tuber storage protein with trypsin inhibitory activity. *Plant Molecular Biology*.

[34] Hou W.-C., Lin Y.-H. (1997). Dehydroascorbate reductase and monodehydroascorbate reductase activities of trypsin inhibitors, the major sweet potato (*Ipomoea batatas* [L.] Lam) root storage protein. *Plant Science*.

[35] Dale M. F. B., Griffiths D. W., Bain H., Todd D. (1993). Glycoalkaloid increase in *Solarium tuberosum* on exposure to light. *Annals of Applied Biology*.

[36] Ginzberg I., Tokuhisa J. G., Veilleux R. E. (2009). Potato steroidal glycoalkaloids: biosynthesis and genetic manipulation. *Potato Research*.

[37] Friedman M. (2006). Potato glycoalkaloids and metabolites: roles in the plant and in the diet. *Journal of Agricultural and Food Chemistry*.

[38] Kuo K.-W., Hsu S.-H., Li Y.-P. (2000). Anticancer activity evaluation of the Solanum glycoalkaloid solamargine: triggering apoptosis in human hepatoma cells. *Biochemical Pharmacology*.

[39] Liu L.-F., Liang C.-H., Shiu L.-Y., Lin W.-L., Lin C.-C., Kuo K.-W. (2004). Action of solamargine on human lung cancer cells-enhancement of the susceptibility of cancer cells to TNFs. *FEBS Letters*.

[40] Ikeda T., Tsumagari H., Honbu T., Nohara T. (2003). Cytotoxic activity of steroidal glycosides from solanum plants. *Biological and Pharmaceutical Bulletin*.

[41] Stahl W., Sies H. (1996). Lycopene: a biologically important carotenoid for humans?. *Archives of Biochemistry and Biophysics*.

[42] Krinsky N. I. (1993). Actions of carotenoids in biological systems. *Annual Review of Nutrition*.

[43] Tapiero H., Townsend D. M., Tew K. D. (2004). The role of carotenoids in the prevention of human pathologies. *Biomedicine and Pharmacotherapy*.

[44] Blanquet-Diot S., MahaSoufi, Rambeau M., Rock E., Alric M. (2009). Digestive stability of xanthophylls exceeds that of carotenes as studied in a dynamic in vitro gastrointestinal system. *Journal of Nutrition*.

[45] Hesam F., Balali G. R., Tehrani R. T. (2012). Evaluation of antioxidant activity of three common potato (Solanum tuberosum) cultivars in Iran. *Avicenna Journal of Phytomedicine*.

[46] Chimkode R., Patil M. B., Jalalpure S. S. (2009). Wound healing activity of tuberous root extracts of *Ipomoea batatas*. *Advances in Pharmacology and Toxicology*.

[47] Panda V., Sonkamble M. (2011). Anti-ulcer activity of *Ipomoea batatas* tubers (sweet potato). *Functional Foods in Health and Disease*.

[48] Suzuki T., Tada H., Sato E., Sagae Y. (1996). Application of sweet potato fiber to skin wound in rat. *Biological and Pharmaceutical Bulletin*.

[49] Dilworth L., Brown K., Wright R., Oliver M., Hall S., Asemota H. (2012). Antioxidants, minerals and bioactive compounds in tropical staples. *African Journal of Food Science and Technology*.

[50] Cornago D. F., Rumbaoa R. G. O., Geronimo I. M. (2011). Philippine Yam (*Dioscorea* spp.) tubers phenolic content and antioxidant capacity. *Philippine Journal of Science*.

[51] Hsu C.-K., Yeh J.-Y., Wei J.-H. (2011). Protective effects of the crude extracts from yam (*Dioscorea alata*) peel on tert-butylhydroperoxide-induced oxidative stress in mouse liver cells. *Food Chemistry*.

[52] Chen Y.-T., Lin K.-W. (2007). Effects of heating temperature on the total phenolic compound, antioxidative ability and the stability of dioscorin of various yam cultivars. *Food Chemistry*.

[53] Chan Y.-C., Chang S.-C., Liu S.-Y., Yang H.-L., Hseu Y.-C., Liao J.-W. (2010). Beneficial effects of yam on carbon tetrachloride-induced hepatic fibrosis in rats. *Journal of the Science of Food and Agriculture*.

[54] Chu Y. H., Chang C. L. (2000). Flavonoid content of several vegetables and their antioxidant activity. *Journal of the Science of Food and Agriculture*.

[55] Brown C. R. (2008). Breeding for phytonutrient enhancement of potato. *American Journal of Potato Research*.

[56] Han K.-H., Shimada K.-I., Sekikawa M., Fukushima M. (2007). Anthocyanin-rich red potato flakes affect serum lipid peroxidation and hepatic SOD mRNA level in rats. *Bioscience, Biotechnology and Biochemistry*.

[57] Ji X., Rivers L., Zielinski Z. (2012). Quantitative analysis of phenolic components and glycoalkaloids from 20 potato clones and in vitro evaluation of antioxidant, cholesterol uptake, and neuroprotective activities. *Food Chemistry*.

[58] Omar N. F., Hassan S. A., Yusoff U. K., Abdullah N. A. P., Wahab P. E. M., Sinniah U. R. (2012). Phenolics, flavonoids, antioxidant activity and cyanogenic glycosides of organic and mineral-base fertilized cassava tubers. *Molecules*.

[59] Panda V., Sonkamble M. (2012). Phytochemical constituents and pharmacological activities of *Ipomoea batatas* l. (Lam)—a review. *International Journal of Research in Phytochemistry & Pharmacology*.

[60] Kim J. J., Kim C. W., Park D. S. (2008). Effects of sweet potato fractions on alcoholic hangover and gastric ulcer. *Laboratory Animal Research*.

[61] Huang D.-J., Lin C.-D., Chen H.-J., Lin Y.-H. (2004). Antioxidant and antiproliferative activities of sweet potato (*Ipomoea batatas* [L.] Lam “Tainong 57”) constituents. *Botanical Bulletin of Academia Sinica*.

[62] Yoshimoto M., Okuno S., Yoshinaga M., Yamakawa O., Yamaguchi M., Yamada J. (1999). Antimutagenicity of sweet potato (*Ipomoea batatas*) roots. *Bioscience, Biotechnology and Biochemistry*.

[63] Thompson M. D., Thompson H. J., McGinley J. N. (2009). Functional food characteristics of potato cultivars (*Solanum tuberosum* L.): phytochemical composition and inhibition of 1-methyl-1-nitrosourea induced breast cancer in rats. *Journal of Food Composition and Analysis*.

[64] Madiwale G. P., Reddivari L., Holm D. G., Vanamala J. (2011). Storage elevates phenolic content and antioxidant activity but suppresses antiproliferative and pro-apoptotic properties of colored-flesh potatoes against human colon cancer cell lines. *Journal of Agricultural and Food Chemistry*.

[65] Auyeung K. K.-W., Law P.-C., Ko J. K.-S. (2009). *Astragalus* saponins induce apoptosis via an ERK-independent NF-*κ*B signaling pathway in the human hepatocellular HepG2 cell line. *International Journal of Molecular Medicine*.

[66] Lee K.-R., Kozukue N., Han J.-S. (2004). Glycoalkaloids and metabolites inhibit the growth of human colon (HT29) and liver (HepG2) cancer cells. *Journal of Agricultural and Food Chemistry*.

[67] Wang T.-S., Lii C.-K., Huang Y.-C., Chang J.-Y., Yang F.-Y. (2011). Anticlastogenic effect of aqueous extract from water yam (*Dioscorea alata* L.). *Journal of Medicinal Plant Research*.

[68] Choi E. M., Koo S. J., Hwang J.-K. (2004). Immune cell stimulating activity of mucopolysaccharide isolated from yam (*Dioscorea batatas*). *Journal of Ethnopharmacology*.

[69] Su P.-F., Li C.-J., Hsu C.-C. (2011). Dioscorea phytocompounds enhance murine splenocyte proliferation ex vivo and improve regeneration of bone marrow cells in vivo. *Evidence-Based Complementary and Alternative Medicine*.

[70] Shang H.-F., Cheng H.-C., Liang H.-J., Liu H.-Y., Liu S.-Y., Hou W.-C. (2007). Immunostimulatory activities of yam tuber mucilages. *Botanical Studies*.

[71] Olayemi J. O., Ajaiyeoba E. O. (2007). Anti-inflammatory studies of yam (*Dioscorea esculenta*) extract on Wistar rats. *African Journal of Biotechnology*.

[72] Kaspar K. L., Park J. S., Brown C. R., MAthison B. D., Navarre D. A., Chew B. P. (2011). Pigmented potato consumption alters oxidative stress and inflammatory damage in men. *Journal of Nutrition*.

[73] Tripathi A. S., Chitra V., Sheikh N. W., Mohale D. S., Dewani A. P. (2010). Immunomodulatory activity of the methanol extract of *Amorphophallus campanulatus* (Araceae) Tuber. *Tropical Journal of Pharmaceutical Research*.

[74] Sonibare M. A., Abegunde R. B. (2012). *In vitro* antimicrobial and antioxidant analysis of *Dioscorea dumetorum* (Kunth) Pax and *Dioscorea hirtiflora* (Linn.) and their bioactive metabolites from Nigeria. *Journal of Applied Biosciences*.

[75] Ludvik B. H., Mahdjoobian K., Waldhaeusl W. (2002). The effect of *Ipomoea batatas* (Caiapo) on glucose metabolism and serum cholesterol in patients with type 2 diabetes: a randomized study. *Diabetes Care*.

[76] Kusano S., Abe H. (2000). Antidiabetic activity of white skinned sweet potato (*Ipomoea batatas* L.) in obese Zucker fatty rats. *Biological and Pharmaceutical Bulletin*.

[77] Ludvik B., Neuffer B., Pacini G. (2004). Efficacy of *Ipomoea batatas* (Caiapo) on diabetes control in type 2 diabetic subjects treated with diet. *Diabetes Care*.

[78] Maithili V., Dhanabal S. P., Mahendran S., Vadivelan R. (2011). Antidiabetic activity of ethanolic extract of tubers of *Dioscorea alata* in alloxan induced diabetic rats. *Indian Journal of Pharmacology*.

[79] Son I. S., Kim J. H., Sohn H. Y., Son K. H., Kim J.-S., Kwon C.-S. (2007). Antioxidative and hypolipidemic effects of diosgenin, a steroidal saponin of yam (*Dioscorea* spp.), on high-cholesterol fed rats. *Bioscience, Biotechnology and Biochemistry*.

[80] Ma H. Y., Zhao Z. T., Wang L. J., Wang Y., Zhou Q. L., Wang B. X. (2002). Comparative study on anti-hypercholesterolemia activity of diosgenin and total saponin of *Dioscorea panthaica*. *China Journal of Chinese Materia Medica*.

[81] Cayen M. N., Dvornik D. (1979). Effect of diosgenin on lipid metabolism in rats. *Journal of Lipid Research*.

[82] Thewles A., Parslow R. A., Coleman R. (1993). Effect of diosgenin on biliary cholesterol transport in the rat. *Biochemical Journal*.

[83] Uchida K., Takse H., Nomura Y., Takeda K., Takeuchi N., Ishikawa Y. (1984). Effects of diosgenin and B-sisterol on bile acids. *The Journal of Lipid Research*.

[84] Zhi-Dong X., Peng-Gao L., Tai-Hua M. (2009). The differentiation- and proliferation-inhibitory effects of sporamin from sweet potato in 3T3-L1 preadipocytes. *Agricultural Sciences in China*.

[85] Temel R. E., Brown J. M., Ma Y. (2009). Diosgenin stimulation of fecal cholesterol excretion in mice is not NPC1L1 dependent. *Journal of Lipid Research*.

[86] Wu W.-H., Liu L.-Y., Chung C.-J., Jou H.-J., Wang T.-A. (2005). Estrogenic effect of yam ingestion in healthy postmenopausal women. *Journal of the American College of Nutrition*.

[87] Chen J.-H., Wu J. S.-S., Lin H.-C. (2008). Dioscorea improves the morphometric and mechanical properties of bone in ovariectomised rats. *Journal of the Science of Food and Agriculture*.

[88] Hwang Y. P., Choi J. H., Han E. H. (2011). Purple sweet potato anthocyanins attenuate hepatic lipid accumulation through activating adenosine monophosphate–activated protein kinase in human HepG2 cells and obese mice. *Nutrition Research*.

[89] Ray R. C., Sivakumar P. S. (2009). Traditional and novel fermented foods and beverages from tropical root and tuber crops: review. *International Journal of Food Science and Technology*.

[90] Ahmed M., Akter M. S., Eun J.-B. (2010). Peeling, drying temperatures, and sulphite-treatment affect physicochemical properties and nutritional quality of sweet potato flour. *Food Chemistry*.

[91] Caperuto L., Amaya-Farfan J., Camargo C. (2000). Performance of quinoa (*Chenopodium quinoa* wild) flour in the manufacture of gluten-free spaghetti. *Journal of the Science of Food and Agriculture*.

[92] Mondy N. I., Gosselin B. Y. (1988). Effect of peeling on total phenols, total glycoalkaloids, discoloration and flavor of cooked potatoes. *Journal of Food Science*.

[93] Sapers G. M., Cooke P. H., Heidel A. E., Martin S. T., Miller R. L. (1997). Structural changes related to texture of pre-peeled potatoes. *Journal of Food Science*.

[94] Shih M.-C., Kuo C.-C., Chiang W. (2009). Effects of drying and extrusion on colour, chemical composition, antioxidant activities and mitogenic response of spleen lymphocytes of sweet potatoes. *Food Chemistry*.

[95] Kampa M., Alexaki V.-I., Notas G. (2004). Antiproliferative and apoptotic effects of selective phenolic acids on T47D human breast cancer cells: potential mechanisms of action. *Breast Cancer Research*.

[96] Wu X., Sun C., Yang L., Zeng G., Liu Z., Li Y. (2008). *β*-Carotene content in sweet potato varieties from China and the effect of preparation on *β*-carotene retention in the Yanshu No. 5. *Innovative Food Science & Emerging Technologies*.

[97] Barba A. A., Calabretti A., d'Amore M., Piccinelli A. L., Rastrelli L. (2008). Phenolic constituents levels in cv. Agria potato under microwave processing. *LWT—Food Science and Technology*.

[98] Takenaka M., Nanayama K., Isobe S., Murata M. (2006). Changes in caffeic acid derivatives in sweet potato (*Ipomoea batatas* L.) during cooking and processing. *Bioscience, Biotechnology and Biochemistry*.

[99] Mondy N. I., Gosselin B. (1989). Effect of irradiation on discoloration, phenols and lipids of potatoes. *Journal of Food Science*.

[100] Navarre D. A., Shakya R., Holden J., Kumar S. (2010). The effect of different cooking methods on phenolics and vitamin C in developmentally young potato tubers. *American Journal of Potato Research*.

[101] Rein M. (2005). *Copigmentation reactions and color stability of berry anthocyanins [Dissertation]*.

[102] Patras A., Brunton N. P., O'Donnell C., Tiwari B. K. (2010). Effect of thermal processing on anthocyanin stability in foods; mechanisms and kinetics of degradation. *Trends in Food Science and Technology*.

[103] Fang Z., Wu D., Yü D., Ye X., Liu D., Chen J. (2011). Phenolic compounds in Chinese purple yam and changes during vacuum frying. *Food Chemistry*.

[104] Chen Y.-T., Kao W.-T., Lin K.-W. (2008). Effects of pH on the total phenolic compound, antioxidative ability and the stability of dioscorin of various yam cultivars. *Food Chemistry*.

[105] USDA NAL https://fnic.nal.usda.gov/food-composition.

